# Trypanocidal Effect of Nano MOFs-EP on Circulating Forms of *Trypanosoma cruzi*

**Published:** 2020

**Authors:** Marisol MORALES-BAEZ, José María RIVERA-VILLANUEVA, Aracely LÓPEZ-MONTEON, Rodolfo PEÑA-RODRÍGUEZ, Ángel TRIGOS, Angel RAMOS-LIGONIO

**Affiliations:** 1. Biomedical Sciences, Veracruzana University, Xalapa, Veracruz, México; 2. LADISER Organic Chemistry, Faculty of Chemical Sciences, Veracruzana University, Orizaba, Veracruz, México; 3. LADISER Immunology and Molecular Biology, Faculty of Chemical Sciences, Veracruzana University, Orizaba, Veracruz, México; 4. Biomedical Research Center, Veracruzana University, Xalapa, Veracruz, México

**Keywords:** Metal-organic frameworks (MOFs), Ergosterol peroxide, Chagas disease, *Trypanosoma cruzi*, Trypanocidal activity

## Abstract

**Background:**

Chagas disease caused by the parasite *Trypanosoma cruzi* is considered a neglected disease in several countries. One of the main problems about this disease is the lack of an effective treatment and the absence of adverse effects. *T. cruzi*, like most pathogenic fungi and yeasts, require specific sterols to maintain viability and proliferative capacity during their life cycle. However, the oxidation of this molecule to ergosterol peroxide has shown several biological effects, including its trypanocidal activity.

**Methods:**

We have synthesized MOFs nanoparticles as carrier system coupled to ergosterol peroxide (MOFs-EP) and we have studied its effect on the circulating forms of the *T. cruzi* parasite.

**Results:**

MOFs-EP possess an efficient trypanocidal activity at much lower inhibitory concentrations (ng/mL) that the concentrations shown by ergosterol peroxide (μg/mL) when administered unconjugated form.

**Conclusion:**

Our results open a new possibility in the biomedical application of MOFs and ergosterol peroxide in the search for new options for the treatment of Chagas disease.

## Introduction

Infectious diseases caused by pathogenic microorganisms are the most prevalent worldwide. Chagas disease (American trypanosomiasis), a potentially deadly parasitic disease caused by the protozoan *Trypanosoma cruzi* (*T. cruzi*), and it remains one of the neglected tropical diseases. According to WHO, around 7 to 8 million people are infected worldwide, mainly in Latin America, where Chagas disease is endemic (1). In relation to the treatment of Chagas disease, the options are benznidazole (BNZ) (*N*-benzyl-2-nitroimidazole-1-acetamide) a nitroimidazole derivative and the nifurtimox (NFX) (4[(5-nitrofurfurylidene) amino]-3-methylthiomorpholine-1,1-dioxide) a derivative of nitrofuran, these treatments are not entirely effective, they are quite nonspecific, possess significant activity only in the acute phase of the disease and cause serious side effects (2, 3).

A new class of hybrid materials called metal-organic frameworks (MOFs) has recently been emerged which possess many potential applications in different areas of the chemistry such as catalysis (4), nonlinear optics (5), gas storage (6), biomedical imaging (7), as carrier systems (8), among others. These coordination polymers can be prepared using self-assembly between a variety of organic ligands and metal ions (9). Furthermore, MOF materials can be prepared in the nanoscale using different methodologies actually reported in the literature (10, 11). One of the principal characteristics of MOF materials is the high surface areas they possess increased in the nanoscale, in-consequent the weak interactions that occurs on the surface of nano MOFs with other molecules are improved as well. Moreover, their unique properties such as high surface areas, crystallinity and tunable porosity provide great potential for functionalization on their internal as well as on their external surface (12–14).

The use of MOF nanoparticles in biomedical applications involves the interaction of the surface with the specific molecules under investigation, for that reason the external surface functionalization must fulfills different tasks (15). The interactions between the nano-particles and different molecules under investigation must be week interactions such hydrogen bonding and Van der Waals. Theoretical studies have already been made to investigate the strength of the hydrogen bonding between the COOH group of Levodopa and the NH _2_ group belonging to a functionalized pillared coordination polymer. The results obtained suggest the possibility to employ such materials as carrier systems because the NH _2_ group can form strong hydrogen-bonding interactions with any electron acceptor groups (16).

Sterols are important structural and functional components of the plasma membranes of eukaryotic cells, in particular, cholesterol, ergosterol and phytosterols associated with plasma membranes of vertebrates, fungi and plant cells, respectively (17). In particular, ergosterol is the precursor of several molecules essential for cells such as vitamin D2 among others (18). Ergosterol is oxidized to form ergosterol peroxide (EP) isolated from a wide variety of fungi; attributed several immunosuppressive, anti-inflammatory, antiviral, anti-tumor, antiparasitic and trypanocidal biological activities in vitro (19, 20, 17). The necessity to focus the research on new trypanocidal molecules is due to the failed search for new drugs against the parasite *T. cruzi*, which causes Chagas disease (21).

In the present study, we have synthesized MOFS nanoparticles as carrier system coupled to ergosterol peroxide and we have studied its effect on the circulating forms of the *T. cruzi* parasite in order to improve and expand its biomedical application that allows the search of new options for the treatment of the Chagas disease.

## Materials and Methods

This work was carried out during the year 2018 in the laboratories; LADISER Organic Chemistry and LADISER Immunology and Molecular Biology of the faculty of chemical sciences, region Orizaba of the Veracruzana University.

### Materials

The heterogeneous catalyst catena-((μ _2 _-5-nitrobenzene-1,3-dicarboxilate-O,O′,O″)-(μ _2-_ 1,2-bis(4-pyridyl)-ethane-N,N′)-Cobalt II was passed through a 260 microns sieve and then activated at 100 °C during 24 h previous to use. All reactants and solvents were purchased from Aldrich Chemical Co. and solvents were dried prior to use (22). Single crystals were grown using spectroscopic grade solvents.

### Instrumentation

Elemental analyses were performed on a PerkinElmer Series II CHNS/O model 2400 analyzer. IR spectra were recorded on a PerkinElmer FTIR/FTFIR Spectrum 400 spectrometer with a reflectance ATR accessory. Single crystal X-ray diffraction analyses of compound (**A)** was performed on an Enraf-Nonius Kappa-CCD (λ MoK _α_ = 0.71073 Å, graphite monochromator, T= 298 K, CCD rotating images scan mode). All reflection data set were corrected for Lorentz and polarization effects.

### Synthesis of MOF nanoparticles (A)

**[Zn (L1)_2_]**_n_ (**A**). A mixture containing Zn (NO_3_)_2_·6H_2_O (100 mg, 0.34 mmol), **L1** (50 mg, 0.27 mmol), **L2** (63.7 mg, 0.30 mmol) H _2_O (20 mL) was heated at 160 °C for 72 h. After the reaction was cooled slowly to room temperature, rectangular shaped colorless crystals were obtained with a yield around 47%. Mp>350 °C. IR υ _max_ : 3250(O-H) 1654 (N=O), 1558 (C=O), 1492 (C=N), 1401, 1326, 1281, 1255, 1143, 1028, 955, 887, 817, 743, 681 cm ^−1^ . Anal. Calc. for C _28_ H _17_ CoN _4_ O _12_ : C, 50.92; H, 2.59; N, 8.48%. Found C, 51.04; H, 2.31; N, 8.68%.

### Synthesis of ergosterol peroxide

The ergosterol peroxide was obtained and characterized according to what was reported by (17), briefly, chemical compound was obtained by sensitized photooxygenation in methanol with eosine as the sensitizer from ergosterol (ergosta-5,7,22-trien-3β-ol) to obtain ergosterol peroxide (EP; 5,8-epidioxy-5α,8α-ergosta-6,22-dien-3β-ol).

### Coupling of ergosterol peroxide to the carrier system Zn-MOF

The coupling of ergosterol peroxide to nMOFs was performed by mechanochemistry. Briefly, from 50 mg of EP and 100 mg of Zn-MOFs, a mixture was made in a mortar for 10 min, until a homogeneous mixture was obtained, then 150 μl of methanol was added and it was continued mixing for 10 min more. The obtained mixture was subjected to a drying process at 105 °C for 30 min, the material obtained was passed through a sieve with an opening of 25 microns. The crystalline powder obtained was analyzed by infrared spectroscopy (IR) (Perkin Elmer spectrum 100 FT-IR), by X-ray diffraction (D2-PHASER, XRD BRUKER coupled to a flash detector by using the programs WinGx (23), Olex2 (http://www.olexsys.org/) and Mercury 3.3 to obtain the structures of the materials and crystallographic data). In addition, the morphology of the nMOFs-EP was analyzed by transmission electron microscopy (TEM) (TEM-1400 Plus Electron Microscope) and by scanning electron microscopy (SEM, QUANTA FEG 250). The MOFs-EP were recovered and stored at 25 °C until their use.

### Cell lines

The mammalian cells NIH-3T3, J774A.1, and VeRo, infected or not, were maintained in Dulbecco’s modified Eagle medium (DMEM; Gibco Invitrogen), pH 7.4, supplemented with 2mM L-glutamine, 10% FBS, and 50 mg/L gentamicin at 37 °C in a humidified 5% CO2 atmosphere. The cells were first grown, then trypsinized and transferred to 24 or 96-well plates, depending on the assay to be performed.

### Parasites

Parasites of the strain TDIM/MEX/2014/LJ01/*T. cruzi* were obtained from a *Triatoma dimidiata* vector captured in a rural endemic of Veracruz, México. Epimastigote forms were axenically cultivated at 28 °C in Liver Infusion Tryptose (LIT) medium supplemented with 10% heat-inactivated fetal bovine serum (FBS; Gibco Invitrogen, Grand Island, NY, USA) and 0.1% hemin, at a temperature of 28 °C, and maintained by weekly transfers. The trypomastigotes were daily collected from the supernatant of infected NIH-3T3 cells and harvested by centrifugation.

### Evaluation of the cytotoxicity of MOFs

The MTT (3-[4,5-dimethylthiazol-2-yl]-2,5-diphenyltetrazolium bromide) assay was applied. VeRo cells were collected from confluent cultures, plated in 96-well plates, and incubated at 37 °C in a humid 5% CO2 atmosphere. After 24 h, the medium was replaced with new DMEM containing increasing concentrations of MOFs. Following 96 h incubation, the cells were washed in PBS, and 50 mL of MTT (2 mg/mL) was added to each well. The formazan crystals were solubilized in DMSO and absorbance was read at 570nm in a microplate reader (BioTekePower Wave XS). The concentration that reduced 50% of the absorbance value observed in the control represented the CC50 (cytotoxic concentration for 50% of the cells). As a positive control of cytotoxicity using 10% SDS.

### Trypanocidal activity

Cultures were initiated with a cell density of 1 × 10 ^5^ trypomastigotes / mL or epimastigotes, as the case may be 200 μL of the parasite suspension, was deposited in a 96-well microplate in triplicate. The parasites were incubated for 24, 48 and 72 h in the presence of different concentrations (5, 10, 20, 50, 100 y 500 ng/mL) of MOFs and MOFs-EP, Suspensions of MOFs were performed in culture medium. The parasites that interacted with the MOFs and with the MOFS-EP were incubated at 28 °C and the viability of the parasites was determined by counting parasites in a Neubauer chamber. Cell viability was monitored by trypan blue exclusion analysis by light microscopy. All experiments were performed in triplicate and independently.

### Statistical analysis

The experiments were performed in three replicates for each condition, and were carried out at least twice to ensure reproducibility. One-way analysis of variance (ANOVA) and Tukey multiple comparison tests were used for statistical analyses. Statistical significance was defined as *P*<0.05. Statistical analyses were performed using Prism 7.0a (GraphPad Software, La Jolla, CA).

## Results

### MOFs and MOFs-PE characterization

**Fig. 1:**
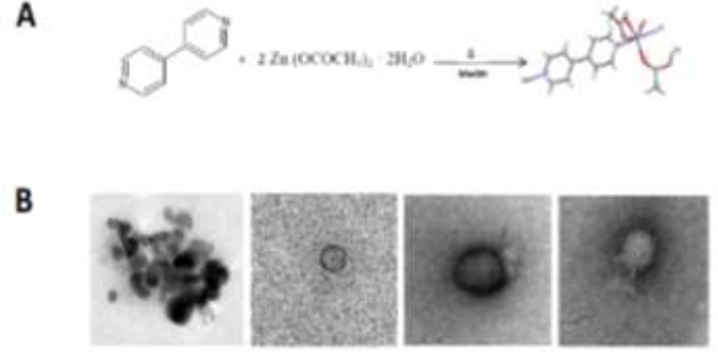
A) Reaction of 4,4′-bipyridyl and zinc acetate dihydrate in methanol, B) TEM of nMOFs-EP

The Zn-MOFs crystallized in a triclinic system within the space group P-1, it turned out to be a zinc (II) polymer formed by two organic linkers, 4, 4′-bipyridyl and acetate (Fig. 1A). The distances of the coordination bonds are in the range from 2.018 to 2.296 Å. Zinc atom is hexacoordinated presenting a distorted octahedral geometry. Acetate groups are coordinated in two ways, one acetate is coordinated as a chelate through both oxygen atoms to the metal, while the second anion acts as a bridging ligand between two zinc atoms. Two acetate bridges lead to the formation of an 8-membered ring. The bond angles around the metal are in the range of 58.60 to 176.55°. The diffraction pattern of MOF and NPsPE obtained by mechanochemical synthesis was analyzed by a XRD analysis of powders using the crystalline powder or Debye-Scherrer method. This analysis generated a diffractogram of the aforementioned molecules, compared with a simulated diffractogram obtained with the Mercury 3.3 program, observing that the diffraction patterns are similar. By means of SEM analysis, the analyzed material shows agglomeration of the particles, with a spherical structure of heterogeneous size that oscillates between 28.67nm – 80.44 nm. On the other hand, the analysis obtained by TEM showed that some particles showed an oval shape but most of the nMOFs-EP (Fig. 1 B) had a circular shape and these particles have sizes less than 100nm, and a surface charge of +31 mV.

### MOFs do not induce cytotoxicity in mammalian cells

**Fig. 2:**
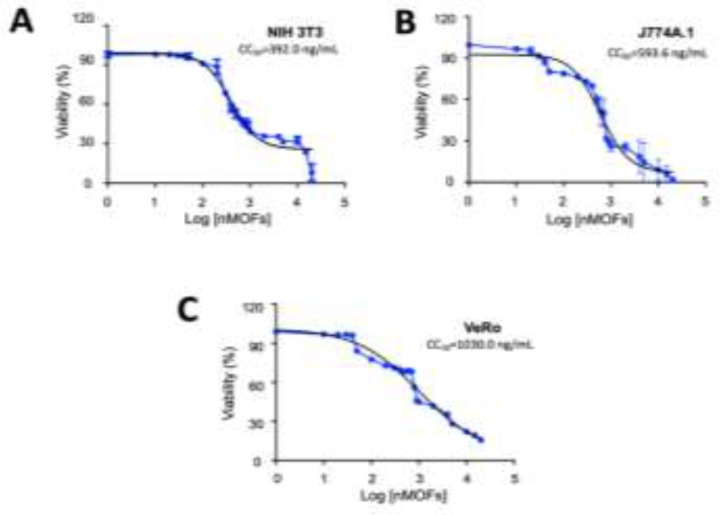
Cytotoxicity assay of MOFs on mammalian cells. A) NIH 3T3, B) J774a.1, and C) VeRo cells. Different concentrations of MOFs were applied to the cell monolayers in triplicate, the cell growth was evaluated by the MTT assay. The 50% cytotoxic concentration (CC50) was defined as the concentration that reduces the optical density (OD570) of treated cells to 50% of untreated cells

To evaluate further the possible toxic effect of MOFs on mammalian cells, NIH-3T3, J774A.1 and VeRo cells were treated with different concentrations of MOFs and after 96 h the viability was assessed using a MTT assay. When the cells were incubated with different concentrations of MOF, no cellular cytotoxicity (CC50) was observed at low concentrations of MOFs, since the OD values obtained from treated and untreated cells were similar. However, when these cells were incubated with 1% Triton X-100, considerable damage to the mono-layer of cells was observed (Data not shown). The MOFs showed a CC50 value of 392.0 ng/mL (IC95% 487.0 to 370.3 ng/mL) for NIH3T3 cells (Fig. 2A), a value of 593.6 ng/mL (IC95%: 681.31 to 592.63 ng/mL) for J774A.1 cells (Fig. 2B), and 1030.0 ng/ml (1011.0 to 1124.9 ng/mL) for VeRo cells (Fig. 2C).

### MOFs-EP have trypanocidal effect

**Fig. 3:**
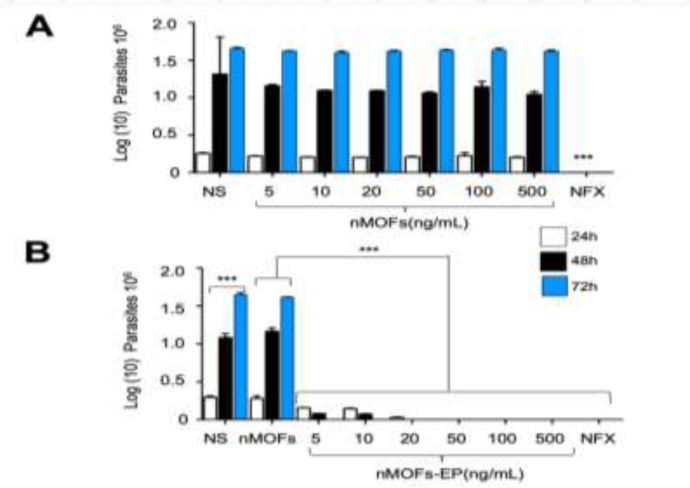
Effect of MOFs-EP on the growth of tripomastigote forms of *T. cruzi*. Tripomastigotes were cultured with different concentrations of MOFs-EP for 24–72h, at the end of which the viability of the parasites on the incubations was determined. Values shown are the means ± SD of triplicate cultures. The data are representative of the results of three independent experiments (*, **, p<0.001 significantly different from the (control) group untreated (NS), treated with MOFs and NFX at 24 and 48h respectively)

To analyze that the trypanocidal effect exerted on the parasites was due to the presence of the EP coupled to the MOFs, the parasites were incubated with different concentrations of MOFs-EP at different times (Fig. 3). The presence of the MOFs at the concentrations used did not affect the growth of the parasite at the incubation times used (Fig. 3A) compared to the effect caused by the positive control (NFX). On the other hand, when the parasites were incubated in the presence of the MOFs-EP, a decrease in growth was observed depending on the concentration and incubation time, in comparison to the growth observed in the culture of parasites incubated with the MOFs alone or without stimulation. The MOFs-EP showed a IC50 value of 4.81 ng/mL (IC95%: 0.57 to 12.46 ng/mL) and 3.0 ng/mL (IC95%: 1.06 to 11.05 ng/mL) for 24 and 48 h respectively. The inhibition observed in the growth of the parasite in the presence of the MOFs-EP was comparable to that observed in the culture of the parasites interacted with NFX (Fig. 3B). In Fig. 3 only the effect on the trypomastigote forms is shown, since the effect observed with the epimastigote forms was very similar (Data not shown).

## Discussion

Despite the efforts made in the treatment of infections caused by parasites, they continue to increase, particularly in tropical and low-income countries, and some of the problems associated with these infections include drug toxicity, inefficiency and development of resistance to conventional drugs, in addition to the costs of treatment (24).

Both NFX and BZN have been widely used for the treatment of Chagas disease and are considered less effective during the chronic phase of infection and the disagreement among scientists regarding its effectiveness during this period and its high rates of adverse effects force the search for new compounds. Whereby, the challenge of controlling and eventually eradicating Chagas disease requires the development of new drugs.

One of the recent class of nanoparticles investigated for chemotherapeutic use are metal-organic frameworks (MOFs) which are hybrid polymers that consist of metal ions or clusters and organic ligands. Here we evaluate in a preliminary way the potential of MOFs coupled to ergosterol peroxide against circulating forms of *T. cruzi* for the development of new treatment strategies for Chagas disease. *T. cruzi*, like most pathogenic fungi and yeasts, require specific sterols to maintain viability and proliferative capacity during their life, cycle and specific inhibitors of ergosterol bio-synthesis show antiproliferative capabilities against this parasite, both in vitro and in vivo (25), and we have shown the use of ergosterol peroxide as a compound with a structure similar to ergosterol which can be a good one anti-*T. cruzi* strategy, due to its selectivity for the plasmatic membrane of the parasite, can be ergosterol peroxide a very effective compound in the treatment of the acute phase of the disease by not showing adverse effects as those obtained with drugs established for the treatment of Chagas disease (17). There have been few studies where nano MOFs have been used in the treatment of infections with obligate intracellular parasites, within the most recent highlights the cytotoxic impacts of the MBMOF nanocomposites on promastigotes, intracellular amastigotes de *Leishmania major* (26).

Currently, great attention has been given to nanomaterials in the administration of drugs due to its highly adjustable shape, size and composition, due to the help of the ligands that can be added or to the increase of the permeability and the retention effect of the coupled compounds, what efficient therapy could achieve and eliminate the side effects of a particular drug (27). Due to their characteristics and the possible additional chemical functionalization, MOFs have attracted a growing interest in the biomedical field in recent years.

The ideal trypanocidal agent must be selective and act on the intra and extracellular forms; quickly, to prevent the evolution towards the chronic phase; its pharmacodynamics must reach effective trypanocidal levels of drug concentration in blood plasma, in biological fluids and in tissues, and must not induce resistance of the parasite to medicament (28). Nevertheless, there are no suitable and effective drugs against the chronic phase, which represents a challenge and makes it necessary to have new alternatives for specific and safe treatment for this phase of the disease. Our results show that the use of MOFs-EP has an inhibitory effect on the circulating forms of *T. cruzi* similar to that previously observed with ergosterol peroxide (17), but with much lower doses of compound and it is not toxic to mammalian cells.

One of the limitations of the study was the lack of a model to evaluate the effect of nano MOFs-EP against the intracellular form of the parasite, However, there is a good candidate for the treatment of Chagas disease in the acute phase, In addition, more tests should be performed with chronic phase models as well as adjusting the doses for use in this stage, in other words, it is necessary to develop a model that increases the selectivity for the infected tissues (especially necessary for the elimination of intracellular forms), mediated by a mechanism that facilitates the release of the compound in the cytoplasm and that also allows to reduce the total doses administered, which would be ideal to improve the results of current therapy against the disease.

## Conclusion

These results open up the possibility of using MOFs-EP as a promising candidate for biomedical application in the treatment of Chagas disease, as a novel and efficient drug administration system. Nevertheless, complementary tests should be performed to analyze the side effects in the administration of these particles or its use in the coupling of previously established compounds for the treatment and improvement of the therapeutic result against Chagas disease, since the lack of treatment for this disease raises the need to seek effective solutions for this public health problem.
